# ﻿ *Neothelaxespileata*, a new species from China (Hemiptera, Sternorrhyncha, Aphididae, Thelaxinae)

**DOI:** 10.3897/zookeys.1076.72802

**Published:** 2021-12-08

**Authors:** Li-Yun Jiang, Xiao-Lu Zhang, Jing Chen, Yi-Fang Zhao, Ge-Xia Qiao

**Affiliations:** 1 Key Laboratory of Zoological Systematics and Evolution, Institute of Zoology, Chinese Academy of Sciences, No. 1–5 Beichen West Road, Chaoyang District, Beijing 100101, China Institute of Zoology, Chinese Academy of Sciences Beijing China; 2 College of Life Science, University of Chinese Academy of Sciences, No. 19, Yuquan Road, Shijingshan District, Beijing 100049, China University of Chinese Academy of Sciences Beijing China

**Keywords:** Aphid, new record, Tibetan plateau

## Abstract

*Neothelaxespileata* Qiao **sp. nov.**, found on *Pileamartinii* (Urticaceae) in China, is described and illustrated. *Neothelaxes* Chakrabarti & Quednau is also a new generic record for China.

## ﻿Introduction

The aphid genus *Neothelaxes* was erected by Chakrabarti and Quednau (1996), with *Neothelaxesviticola* Chakrabarti & Quednau, 1996 as the type species. The generic diagnosis is based on the dorsal body setae arranged in single rows, not in groups, and the presence of minute wax gland pores on the sclerites. At present, there are only two known species, *Neothelaxesparthenocissi* Chakrabarti & Quednau, 1996 and *N.viticola* (Blackman and Eastop 2020; Favret 2020). Recently, some unusual specimens were collected on *Pileamartinii* (H. Lev) Hand-Mazz. (Urticaceae) in the Tibetan plateau, China, and they are here described as a new species, *Neothelaxespileata* Qiao sp. nov. The genus *Neothelaxes* is newly recorded from China.

## ﻿Materials and methods

The procedure used for processing and preparing the aphid specimens for microscopic study followsed that of Jiang et al. (2016). The descriptions and drawings provided here were produced from slide-mounted specimens using a Leica DM4000B with a drawing tube attached. The photomicrographic images were prepared with a Leica DM2500 using DIC illumination and processed with the Automontage and Photoshop software.

Aphid terminology in this paper generally follows that of Chakrabarti and Quednau (1996). The unit of measurement is millimetres (mm). The holotype and seven paratypes are deposited in the National Zoological Museum of China, Institute of Zoology, Chinese Academy of Sciences, Beijing, China (**NZMC**), and one paratype is deposited in the Natural History Museum, London, UK (**NHMUK**).

## ﻿Taxonomy

The Thelaxinae (sensu Remaudière and Stroyan 1984) is a small group of aphids, at present comprising the genera *Thelaxes* Westwood, *Glyphina* Koch, *Kurisakia* Takahashi, and *Neothelaxes* Chakrabarti & Quednau. Species of the first three genera are associated with woody trees: *Thelaxes* includes oak-feeding species (Fagaceae), *Glyphina* species are associated with *Alnus* and *Betula* (Betulaceae), and *Kurisakia* species are associated with Juglandaceae and Fagaceae. On the other hand, *Neothelaxes* is recorded only on climbing woody rattan or herbaceous plants such as *Parthenocissus* (Vitaceae) (Chakrabarti & Quednau, 1996) and *Pileamartinii* (Urticaceae). Among the four genera, only *Neothelaxes* is covered with dorsal waxy plates. This genus has a restricted distribution, with the previously-described species occurring only in the Indian Northwest Himalaya and the new species restricted to the southern Tibetan plateau. Species of the genus are probably endemic to the region.

### ﻿ Neothelaxes pileata

Taxon classificationAnimaliaHemipteraAphididae

Qiao sp. nov.

D5FFA370-6B82-5660-A2A4-EB47A625A5B3

http://zoobank.org/FE1B5156-4669-4410-9703-85D3D33C1834

Figures 1–11 , 12–22 , 23–28 , Table 1

#### Specimens examined.

***Holotype***: apterous viviparous female, **China**: Tibet Autonomous Region (Linzhi City: Motuo County, 29.697°N, 95.556°E, altitude 2678 m), 25 July 2019, No. 46755-1-1-1, on *Pileamartinii* coll. X.L. Zhang. ***Paratypes***: 2 apterous viviparous females and 3 first instar nymphs with the same collection data as the holotype; 1 apterous viviparous female, No. 46755-1-2, with the same collection data as the holotype (NHMUK); 2 fourth instar apterous nymphs, China: Tibet Autonomous Region (Linzhi City: Motuo County, 29.697°N, 95.556°E, altitude 2678 m), 17 June 2021, No. 51097-1-1-1, on Pileamartinii coll. M. Qin and X.L. Zhang.

#### Etymology.

The specific name *pileata* is an adjective based on the feminine generic name of the host plant.

#### Description.

Apterous viviparous female: Body small, oval (Figs 12, 23). Adults light dirty green, or dirty yellowish green, nymphs yellowish green, covered with white waxy powder in life (Fig. 23), and found in irregularly spherical galls on the leaves of the host plant. For morphometric data see Table 1.

**Table 1.** Morphometric data for apterous viviparous females of *Neothelaxespileata* Qiao sp. nov. (n = 4, with means in brackets), the measurements are given in mm.

Characters	Apterous viviparous females (n = 4)
Body length	1.260–1.320 (1.288)
Body width	0.820–0.940 (0.868)
Antenna	0.359–0.418 (0.397)
Antennal segment I	0.052–0.064 (0.058)
Antennal segment II	0.047–0.059 (0.053)
Antennal segment III	0.094–0.119 (0.110)
Antennal segment IV	0.054–0.062 (0.059)
Base of antennal segment V	0.082–0.092 (0.087)
Processus terminalis	0.027–0.035 (0.030)
Ultimate rostral segment	0.104–0.109 (0.105)
Hind femur	0.257–0.319 (0.284)
Hind tibia	0.270–0.324 (0.295)
Second hind tarsal segment	0.079–0.084 (0.082)
Siphunculus	0.012–0.022 (0.015)
Basal width of siphunculus	0.054–0.069 (0.058)
Distal width of siphunculus	0.025–0.027 (0.025)
Cauda	0.015–0.037 (0.030)
Basal width of cauda	0.099–0.109 (0.101)
Basal diameter of antennal segment III	0.020–0.025 (0.022)
Widest width of hind femur	0.054–0.057 (0.056)
Width of hind tibia at mid length	0.032–0.040 (0.036)
Longest dorsal cephalic seta	0.017–0.022 (0.021)
Longest marginal seta on abdominal tergite I	0.012 (0.012)
Longest seta on abdominal tergite VIII	0.012–0.015 (0.014)
Longest seta on antennal segment III	0.010–0.020 (0.015)
Longest seta on hind tibia	0.020–0.027 (0.022)

#### Mounted specimens.

Body dorsum pale brown (Fig. 12). Antennae, legs, cauda, anal plate, and genital plate brown, siphunculi and apex of rostrum dark brown. Head to abdominal segment VII fused, sometimes with intersegmental boundary on spinal area between head and pronotum and pronotum and mesonotum, and on spino-pleural areas of abdominal tergites; abdominal segment VIII free (Fig. 12). Dorsal setae of body spine-like (Figs 6, 7). Wax plates large, with many minute wax pores (Figs 15, 17–19). Vertex with one pair of wax plates, pronotum to abdominal tergites I–VII each with one pair of spinal and one pair of marginal wax plates, tergite VIII with a spino-pleural wax plate (Figs 15, 17–19) . Spiracles small and round, spiracular plates small and oval, brown.

**Figures 1–11.**
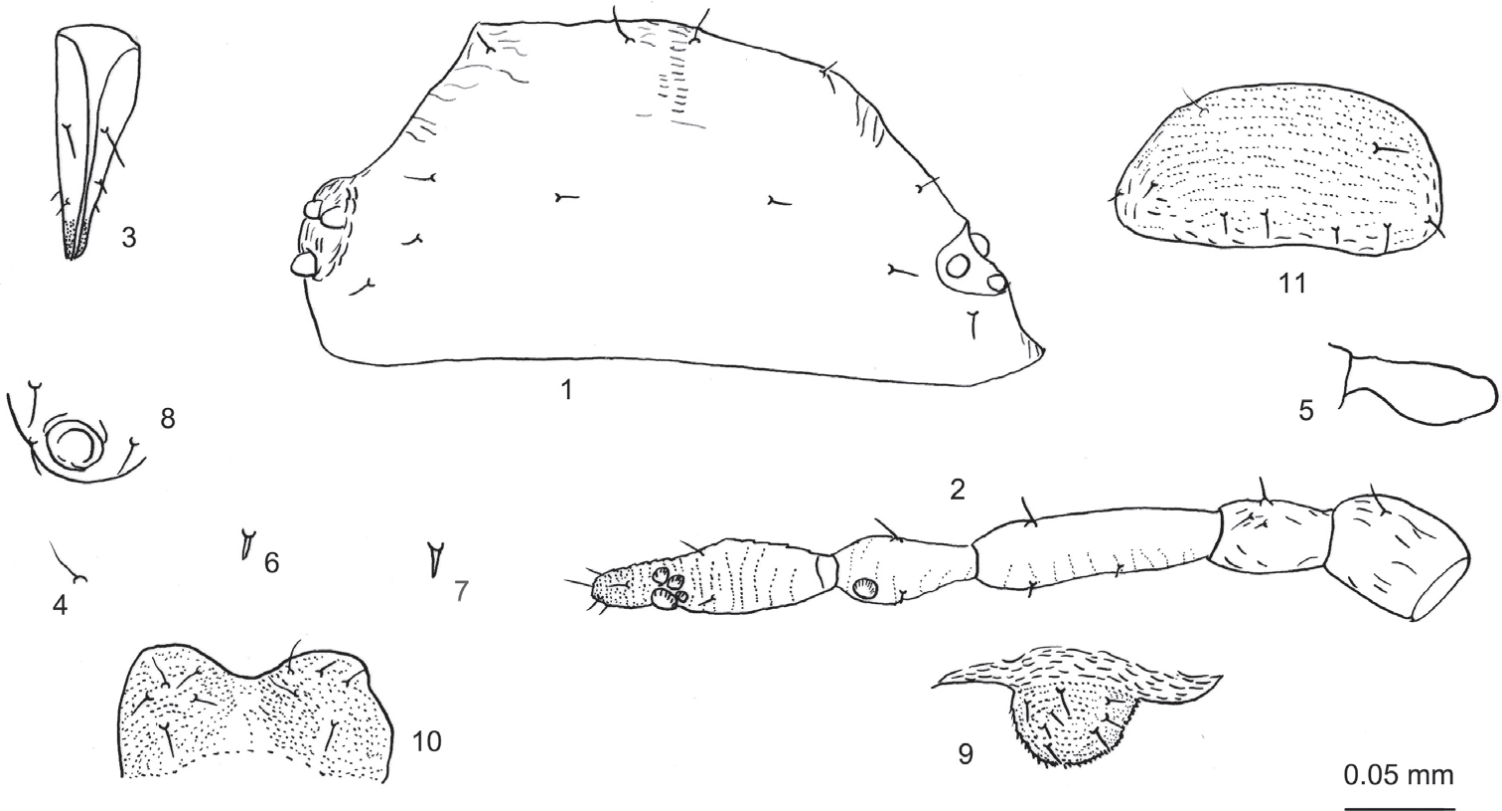
*Neothelaxespileata* Qiao sp. nov. Apterous viviparous female: **1** dorsal view of head **2** antennal segments I–V **3** ultimate rostral segment **4** cephalic seta **5** mesosternal furca **6** marginal seta on abdominal tergite I **7** spinal seta on abdominal tergite VIII **8** siphunculi **9** cauda **10** anal plate **11** genital plate. Scale bars: 0.05 mm.

**Figures 12–22.**
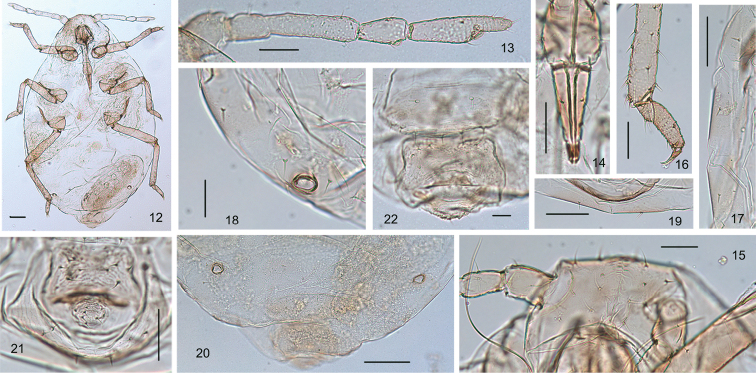
*Neothelaxespileata* Qiao sp. nov. Apterous viviparous female: **12** dorsal view of body **13** antenna **14** ultimate rostral segment **15** dorsal view of head, with antennal segments I–II and dorsal setae **16** distal part of hind tibia and hind tarsal segment **17** marginal setae and marginal waxy plates on abdominal tergites II–V **18** siphunculus and marginal setae and marginal waxy plate on abdominal tergite V **19** spinal setae and waxy plates on abdominal tergite VII **20** siphunculus and dorsal view of abdominal tergites VI–VIII **21** dorsal setae on abdominal tergite VIII, cauda, and anal plate **22** cauda, anal plate and genital plate. Scale bars: 0.10 mm (**12, 17**); 0.05 mm (**13–16, 19–21**); 0.02 mm (**18, 22**).

***Head*.** Frons convex, eyes 3-faceted (Figs 1, 12, 15). Head dorsum with indistinct median suture. Dorsal setae on head short, fine and pointed (Figs 1, 15). Cephalic setae with two pairs, head with one pair of posterior spinal setae and three pairs of marginal setae; cephalic setae 0.78–1.13× basal diameter of antennal segment III (Figs 1, 4, 15). Eyes 3-faceted. Antennae 5-segmented (Figs 2, 13), segments III and IV with sparse spinulose imbrications, segment V with spinulose imbrications; 0.29–0.33× body; processus terminalis 0.30–0.40× base of the segment. Antennal setae sparse, very short and pointed; segments I–V with 2 or 3, 2, 1–4, 3, 2 setae, respectively; processus terminalis with five setae. Length of setae on segment III 0.50–0.80× basal diameter of the segment. Primary rhinaria ciliated (Figs 2, 13). Rostrum (Figs 3, 14) reaching mid-coxae; ultimate rostral segment elongate wedge-shaped, stout at apex, 2.50–3.14× its basal width, 1.18–1.33× second hind tarsal segment, with two pairs of primary setae and two accessory setae, accessory setae longer than primary setae.

***Thorax*** (Fig. 12). Pronotum with one pair of posterior spinal setae and two pairs of marginal setae; meso- and metanotum each with two pairs of marginal setae. Mesosternal furca with two arms separated (Fig. 5). Legs normal. Femur and trochanter fused (Fig. 12); hind femur and trochanter 4.52–5.61× widest width of this segment; 2.41–2.74× antennal segment III. Distal 1/3 of tibiae slightly expanded, with spinulose transverse stripes (Fig. 16); hind tibia 0.21–0.25× body. Setae on legs fine and pointed, length of setae on hind tibiae 0.43–0.73× middle diameter of the segment. First tarsal segments spinulose, segment II with spinulose short stripes (Fig. 16). First tarsal chaetotaxy: 4, 4, 2 or 3.

***Abdomen*.** Abdominal tergites with two or three pairs of spinal and one pair of marginal setae; tergite VII with one pair of spinal and one pair of marginal setae (Fig. 19); tergite VIII with one pair of spinal and two pairs of marginal setae (Fig. 21). Length of marginal setae on tergite I 0.50–0.63× basal diameter of antennal segment III; dorsal setae on tergite VIII 0.56–0.75× basal diameter of antennal segment III. Siphunculi almost poriform (Figs 8, 18, 20), on tergite VI surrounded by three hair-like setae; 0.23–0.32× its basal width, 0.42–0.60× cauda. Cauda knob-shaped (Figs 9, 21–22), with spinulose short stripes; 0.27–0.38× its basal width, with six to eight long and short, finely pointed setae. Anal plate transversely oval (Figs 10, 21–22), indistinctly bilobed, with spinulose short stripes. Genital plate (Figs 11, 22) transverse oval, with sparse spinulose transverse lines; with two anterior setae and seven or eight posterior setae. Two gonapophyses, each with five shorter and pointed gonosetae.

**Figures 23–28.**
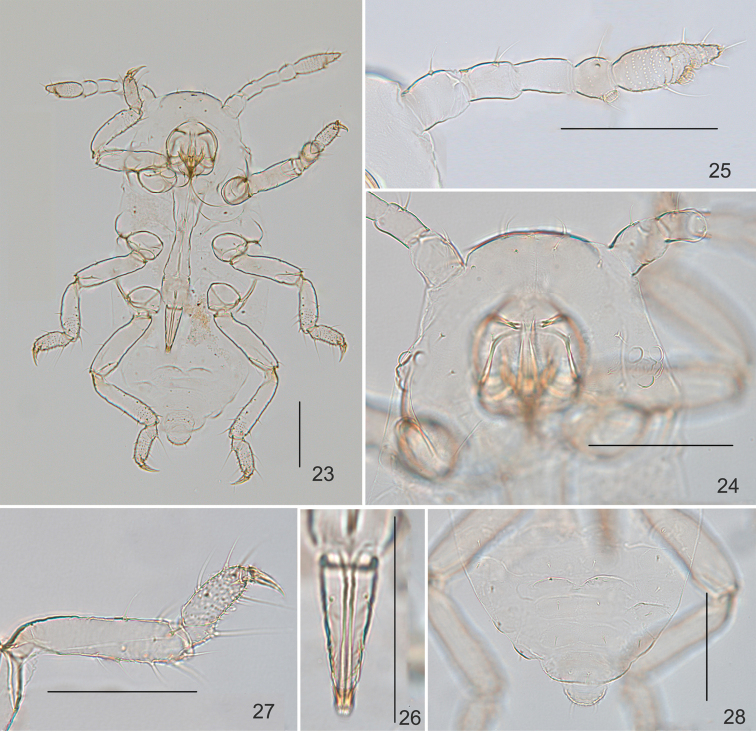
*Neothelaxespileata* Qiao sp. nov. First instar nymph: **23** dorsal view of body **24** dorsal view of head, with antennal segments I–II and dorsal setae **25** antenna **26** ultimate rostral segment **27** hind tibia and hind tarsal segment **28** dorsal setae and waxy plates on abdominal tergites V–VIII, showing cauda. Scale bars: 0.10 mm.

***First instar nymph***: Body oval, pale when macerated. Head and pronotum fused (Fig. 23). Vertex arc-shaped, head dorsum smooth, with distinct median suture (Fig. 24). Dorsal setae on head short and pointed, head with one pair of cephalic setae, two pairs of setae between antennae, three pairs of marginal setae and one pair of anterior spinal setae between eyes; length of cephalic setae 0.83× basal diameter of segment III. Eye 3-faceted. Antennae 5-segmented (Fig. 25), segments I–IV smooth, segment V with spinulose imbrications; antennal setae slightly long and pointed, segments I–V each with 2, 2, 0, 2–3, 2+5 setae, respectively; length of setae on segment IV 1.0× basal diameter of antennal segment III; segment III 0.032 mm, respective length in proportion of segments I–V as follows: 100, 100, 100, 77, 154+77; processus terminalis 0.50× base of the segment. Primary rhinaria round and ciliated. Rostrum reaching abdominal segment IV; ultimate rostral segment elongate wedge-shaped (Fig. 26), 2.82× its basal width, 1.35× hind second tarsal segment; with one pair of accessory setae and two pairs of primary setae. Dorsal setae of thorax and abdomen spine-like, similar to adults. Thorax dorsum each with one pair of spinal and one pair of marginal wax plates, respectively. Pronotum with one pair of spinal and one pair of marginal setae, mesonotum and metanotum each with one pair of spinal, one pair of pleural and two pairs of marginal setae. Trochanter fused with femur. Distal half of tibiae and tarsi with spinulose stripes, the other half of tibiae smooth (Fig. 27). First tarsal chaetotaxy: 2, 2, 2. Abdominal tergites I–VII each with one pair of spinal and one pair of marginal wax plates (Fig. 28); tergite VIII covered with wax plate (Fig. 28). Abdominal tergites I–VII each with one pair of spinal and one pair of marginal setae (Fig. 28); tergite VIII with two dorsal setae (Fig. 28); length of marginal setae on tergite I and dorsal setae on tergite VIII 0.83× and 0.33× basal width of antennal segment III, respectively. Siphunculi invisible. Cauda circular at apex, with two setae (Fig. 28). Anal plate broadly circular, with four setae (Fig. 23). Cauda and anal plate with spinules.

***Embryo* (in an aptera)**: Eye 3-faceted. Antenna 5-segmented, segments I–IV smooth, segment V with spinulose imbrications. Frontal setae hair-like, the remainder of dorsal body setae stout, acute, almost spine-like. Vertex on each side with three anterior and two posterior setae. Pronotum with three pairs of marginal and one pair of spinal setae, anterior spinal setae missing. Meso- and metanotum each with two pairs of marginal, one pair of spinal, and one pair of pleural setae. Abdominal tergites I–VII each with one pair of spinal and one pair of marginal setae; tergite VIII with one pair of dorsal setae. Siphunculi hardly visible. Antennal segments I–IV with 2, 2, 0, 2 or 3 and 2+4 setae, respectively.

#### Host plant.

*Pileamartinii* (H. Lev.) Hand-Mazz. (Urticaceae).

#### Biology.

The specimens were found within an irregularly spherical gall on the leaves. Compared to the other two species in *Neothelaxes*, which are not known to form galls, the biology of this new species is unusual, interesting, but less well known.

#### Comments.

According to some morphological features–3-faceted eye in apterae; fused head and thorax; 5-segmented antenna; processus terminalis shorter than base of the segment; antennal segment V, tarsi, and apices of tibiae spiculose; siphunculi poriform and surrounded by setae; cauda knob-shaped–the new species is regarded as belonging to the subfamily Thelaxinae. This new species is similar to those of *Neothelaxes* based on dorsum of body with waxy plates, dorsal body setae short and spine-like, and primary rhinaria ciliated. However, it differs from the type species of the genus, *N.viticola*, as follows: first tarsal segment chaetotaxy: 4, 4, 2 or 3 (in *N.viticola* first tarsal segments with 5-5-7 setae); dorsum of body pale brown, without distinct sclerites (in *N.viticola* vertex and spinal, marginal, and pleural sclerites of body dorsum distinct); antennae at most 1/3 of body length (in *N.viticola* 1/2 of body length); antennae of embryo 5-segmented (in *N.viticola* 4-segmented); infesting plants of *Pilea* (Urticaceae) (*N.viticola* infests the genus *Parthenocissus* (Vitaceae)).

Of the four known genera of Thelaxinae (sensu Remaudière and Stroyan 1984), *Thelaxes*, *Glyphina*, and *Kurisakia* are associated with woody trees (Fagaceae, Betulaceae, Juglandaceae), wheras *Neothelaxes* is known only from climbing woody rattan (Vitaceae: *Parthenocissus*) (Chakrabarti & Quednau, 1996). No species of Thelaxinae was previously known to live in galls. The new species is associated with an herbaceous plant and was found in leaf galls. These traits are very different from those of other species of Thelaxinae. The association with the galls is unusual and needs further confirmation from a full colony of aphids in a gall.

In view of the present findings on its host association and gall inducing nature as well as several other characters, the new species is placed in the genus *Neothelexes*. Further surveying and research on its biology, for example the rearing of additional adults (especially alatae) from additional galls, will be necessary to elucidate the appropriate taxonomic placement of the new species.

## Supplementary Material

XML Treatment for Neothelaxes pileata
